# Supplementary material for: Complexome profiling of the Chlamydomonas psb28 mutant reveals TEF5 as an early PSII assembly factor
Source: Plant Cell. 2025 Mar 17;37(6):koaf055. doi: 10.1093/plcell/koaf055 (PMC12164590; doi:10.1093/plcell/koaf055)
Supplement: koaf055_Supplementary_Data [file koaf055_supplementary_data.zip › psb28_interactive.zip!/plots/intensity/qualityPlots/peptides/CRT2_Cre01.g038400.t1.2.html]

### Cre01.g038400.t1.2

Trivial name: CRT2  
SeqCoverage [median]: 26.00  
Mass: 47.33 kDa  
  
 

| Sequence | MissedCleavage | Mass [Da] | wtIntensity | muIntensity | Score |
| --- | --- | --- | --- | --- | --- |
| DTVVQFSVK | 0 | 1021.54 | 0.00 | 2698.46 | 85.21 |
| EEEESGHDEL | 0 | 1172.45 | 312.97 | 344.31 | 70.00 |
| FAEDTWGK | 0 | 952.43 | 3334.73 | 3347.78 | 89.30 |
| FVGFELWQVK | 0 | 1251.67 | 1289.31 | 6925.53 | 91.87 |
| GIQTGPDSK | 0 | 901.45 | 850.37 | 3373.51 | 93.37 |
| IPKEEEESGHDEL | 1 | 1510.68 | 9512.40 | 15668.59 | 140.45 |
| KVHVILTYK | 1 | 1099.68 | 1924.16 | 4561.56 | 135.55 |
| SGSIFDNILVTDDLEAAKK | 1 | 2035.05 | 5110.38 | 0.00 | 87.16 |
| VHVILTYK | 0 | 971.58 | 0.00 | 533.29 | 98.30 |
| VSDGSAGEFK | 0 | 995.46 | 8888.31 | 20056.70 | 146.27 |
| VSDGSAGEFKLTAGK | 1 | 1465.74 | 0.00 | 2872.53 | 49.93 |
| VVPATSEK | 0 | 829.45 | 13345.91 | 20220.72 | 98.52 |
| WYGDAEADKGIQTGPDSK | 1 | 1936.88 | 3145.39 | 8789.54 | 91.40 |
|  |  |  | 47713.9 | 89392.5 |  |
